# Prediction of near‐term climate change impacts on UK wheat quality and the potential for adaptation through plant breeding

**DOI:** 10.1111/gcb.16552

**Published:** 2022-12-23

**Authors:** Nick S. Fradgley, James Bacon, Alison R. Bentley, Germano Costa‐Neto, Andrew Cottrell, Jose Crossa, Jaime Cuevas, Matthew Kerton, Edward Pope, Stéphanie M. Swarbreck, Keith A. Gardner

**Affiliations:** ^1^ NIAB Cambridge UK; ^2^ Met Office Exeter UK; ^3^ International Maize and Wheat Improvement Center (CIMMYT) Carretera México‐Veracruz Mexico; ^4^ Institute for Genomics Diversity, Cornell University Ithaca NY USA; ^5^ Universidad Autonoma del Estado de Quintana Roo Chetumal Quintana Roo Mexico; ^6^ DSVUK, Top Dawkins Barn Banbury UK

**Keywords:** adaptation, climate change impacts, genomic prediction, grain quality, wheat breeding

## Abstract

Wheat is a major crop worldwide, mainly cultivated for human consumption and animal feed. Grain quality is paramount in determining its value and downstream use. While we know that climate change threatens global crop yields, a better understanding of impacts on wheat end‐use quality is also critical. Combining quantitative genetics with climate model outputs, we investigated UK‐wide trends in genotypic adaptation for wheat quality traits. In our approach, we augmented genomic prediction models with environmental characterisation of field trials to predict trait values and climate effects in historical field trial data between 2001 and 2020. Addition of environmental covariates, such as temperature and rainfall, successfully enabled prediction of genotype by environment interactions (G × E), and increased prediction accuracy of most traits for new genotypes in new year cross validation. We then extended predictions from these models to much larger numbers of simulated environments using climate scenarios projected under Representative Concentration Pathways 8.5 for 2050–2069. We found geographically varying climate change impacts on wheat quality due to contrasting associations between specific weather covariables and quality traits across the UK. Notably, negative impacts on quality traits were predicted in the East of the UK due to increased summer temperatures while the climate in the North and South‐west may become more favourable with increased summer temperatures. Furthermore, by projecting 167,040 simulated future genotype–environment combinations, we found only limited potential for breeding to exploit predictable G × E to mitigate year‐to‐year environmental variability for most traits except Hagberg falling number. This suggests low adaptability of current UK wheat germplasm across future UK climates. More generally, approaches demonstrated here will be critical to enable adaptation of global crops to near‐term climate change.

## Graphical abstract

Wheat bread‐making quality is strongly affected by the environment, both directly and through genotype by environment interaction (G × E) effects. Augmentation of genomic prediction models with environmental covariables enabled successful G × E prediction in a large historical field trial dataset. Prediction models were projected into future UK climate scenarios. Negative impacts due to increased summer temperatures were predicted in the main wheat growing area in the east, but the climate in the North and South‐west may become more favourable. For most traits, only limited potential was found for breeders to exploit predictable G × E and select stable genotypes to mitigate year‐to‐year climate variability.

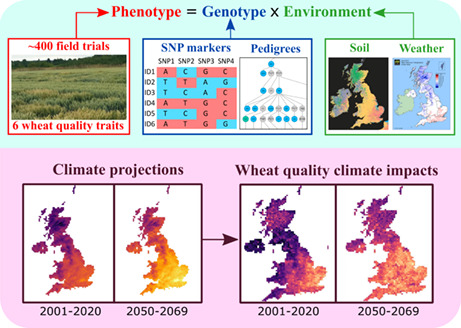

## 1 INTRODUCTION

Climate change threatens crop yields on a global scale (Zhao et al., 2017) and there is a need to develop new climate resilient crop varieties through breeding programmes. Although projected changes in climate may remain favourable for wheat (*Triticum aestivum* L.) yield in the United Kingdom (UK) by the end of the century (Harkness et al., 2020; Slater et al., 2022), impacts on wheat grain quality remain unclear. Anticipating climate change impacts on grain quality and food availability will enable appropriate mitigations and responses.

Wheat quality criteria determine whether grain is suitable for human consumption end uses, such as milling wheat for bread making. These specific quality trait criteria include: high grain specific weight (a volumetric measurement of grain packing density), grain protein content at around 13%, high gluten strength determined using rheological tests such as Chopin alveograph (Bennett & Coppock, 1952) and Zeleny sedimentation (Zeleny et al., 1960), and the degree of starch breakdown resulting from enzyme activity, which is determined using the Hagberg falling number (HFN) test (Perten, 1964). Together, these traits are partly predictive of milling and baking performance (Andersson et al., 1994; Fradgley et al., 2022) determining quality class and value of wheat off‐farm, and are therefore critical traits for selection in wheat breeding. As well as genetic effects that can be selected through breeding, environmental conditions also affect wheat quality traits. For example, HFN is highly sensitive to excessive moisture and low temperatures before harvest (Mares, 1993). Critically, there are often interactions between genotypic and environmental effects (G × E), which means that differences in performance among wheat varieties are partly dependent on the growing environment. These interactions are complex, difficult to predict and hinder genetic gain from breeders' selection (Kang, 1997). Breeding crop varieties adapted to future environments requires both breeding methodologies that can accurately predict G × E and combine this knowledge with plausible future environmental scenarios from climate model projection (Ramirez‐Villegas et al., 2020).

In modern crop breeding programmes, genome‐based prediction of phenotypic values for new genotyped breeding lines allows selections to be made on genetic values (Meuwissen et al., 2001), and has enabled accelerated genetic improvement in many productivity and sustainability traits (Crossa et al., 2017; Voss‐Fels et al., 2019). As well as considering genetic marker effects, recently developed prediction models incorporate environmental co‐variables to characterise their effects on crop phenotypes and further increase the prediction accuracy of breeding lines in particular environments by predicting G × E (Costa‐Neto & Fritsche‐Neto, 2021; Granato et al., 2018; Jarquín et al., 2014). This explicit integration of environmental and genomic information enables novel applications for genomic prediction (Crossa et al., 2021), such as improving the ability to predict performance in untested growing conditions (Costa‐Neto & Fritsche‐Neto, 2021; Guo et al., 2020), optimization of multi‐environment trials networks, and screening genotype‐specific reaction‐norms (Ly et al., 2018; Millet et al., 2019). Costa‐Neto, Fritsche‐Neto, et al. (2021) and Costa‐Neto, Galli, et al. (2021) recently provided a framework for integrating ‘envirotyping’ approaches with genomic prediction models for genotype–environment combinations that are observed in the field. Once reliable prediction is possible, knowledge about G × E can be exploited for adapting crops to specific environmental conditions, or for selecting broadly adaptable varieties that are resilient to variable environments. A method to learn G × E for wheat yield from a large set of observed field trials and make predictions of genotype adaptation and stability in a wider set of historical environments was outlined by de los Campos et al. (2020). Here, we extend this approach by making predictions for a larger number of genotypes across a much larger number of simulated environments derived from future climate projections. We consider grain quality traits which are costly and difficult to measure at high‐throughput, and so are highly valuable to predict.

Large, long‐term and multi‐environment data sets are required to effectively characterise G × E (Jarquín et al., 2014; Poland, 2015). In the UK, several such crop datasets are available from continuously running trial series in historical national variety testing, and as part of plant breeding programmes. The aims of this study were (1) to determine the overall effects of predicted trends in climate change on UK wheat quality using enviromics‐based genomic prediction methods and climate model projections and (2) to quantify the potential for breeding to mitigate adverse effects of year‐to‐year environmental variation by exploiting predictable G × E. We leveraged multiple datasets including several quality traits from UK variety testing and research field trials over 20 years. Soil and weather environmental covariates characterising each trial environment were combined with genetic markers and pedigree data characterising relationships among the wheat genotypes (commercial varieties or breeding lines). We developed genomic prediction models that were able to predict G × E and explain a large proportion of the variation in the data set. We then used these models to predict the quality performance of wheat genotypes in future environments as projected by climate models for 2050–2069 under the Representative Concentration Pathways (RCP) 8.5 emissions pathway.

## 2 METHODS

### 2.1 Multi‐environment trial data

The large‐scale multi‐environment dataset used in this study consists of 392 wheat variety field trial environments (year/location combinations) in the UK spanning 20 years. Of these trials, 323 were part of the Agriculture and Horticulture Development Board (AHDB) Recommended List national variety testing series between harvest years 2001 and 2020. Additional trials included evaluation of 19 high quality milling wheat varieties as part of 32 National Institute of Agricultural Botany (NIAB) membership trials between the harvest years 2009 and 2019, and trials from 2017 to 2019 described in Fradgley et al. (2022) and Scott et al. (2021), which included founders of multi‐parent mapping populations that were varieties in common with AHDB Recommended List trials. Finally, data for nine advanced breeding line evaluation trials as part of the breeding programme from the UK branch of the commercial breeding company Deutsche Saatveredelung (DSV) including 40 lines between 2017 and 2019 were also used. All field trials were managed according to current best practices and economic optimum fertiliser rates for specific field environments (AHDB, 2022b). The field trials are representative of the major UK wheat growing regions.

In total, trial data for 240 genotypes (varieties and breeding lines) were used for which pedigree and/or genetic marker data were also available. The wheat quality traits, grain protein content (%), Hagberg falling number (HFN; seconds) and specific weight (kg hl^−1^), were all measured at between 313 and 327 trial environments. Gluten quality traits: Zeleny sedimentation volume, Chopin alveograph *W* and *P*/*L* values, were measured at between 81 and 86 environments and for fewer genotypes (Table 1). Grain protein content quantifies the percentage of grain weight that is made up of all protein. HFN quantifies the alpha amylase activity of milled flour as the time taken for a plunger to fall through a heated solution of flour and water undergoing gelatinisation. Higher falling numbers indicate lower amylase activity and higher starch quality. Specific weight quantifies the volumetric density of a grain sample as indication of grain quality and suitability for milling. The Chopin alveograph method involves inflating a bubble of dough and measuring the pressure over a time period. The *W* parameter indicates gluten strength as the area under the pressure curve required to inflate and burst a dough bubble while *P*/*L* indicates gluten extensibility as the ratio between the peak pressure and the length of time taken for the bubble to burst.

**TABLE 1:** The number of genotypes tested in each trial year for each trait and the total number of genotypes tested for each trait across the entire trial dataset

Number of genotypes tested per year																					Total genotypes tested
	2001	2002	2003	2004	2005	2006	2007	2008	2009	2010	2011	2012	2013	2014	2015	2016	2017	2018	2019	2020	
Protein	0	25	34	36	38	34	34	52	33	39	39	43	49	43	48	46	63	87	45	23	234
HFN	0	25	34	36	38	34	34	52	33	39	39	43	49	43	48	46	60	75	45	23	232
Specific weight	0	25	35	35	38	34	34	52	33	39	39	43	49	43	48	46	60	86	66	23	237
Zeleny	0	19	2	12	21	1	9	15	1	7	0	0	4	11	16	9	10	12	8	6	93
Chopin *W*	2	18	7	0	0	6	15	19	19	9	9	7	5	18	16	16	10	12	8	6	88
Chopin *P*/*L*	2	18	7	0	0	6	15	19	19	6	9	7	5	18	16	16	10	12	8	6	88
Number of trials per year	3	18	19	15	17	21	21	18	20	26	20	24	15	20	21	20	24	23	29	15	

**FIGURE 1:**
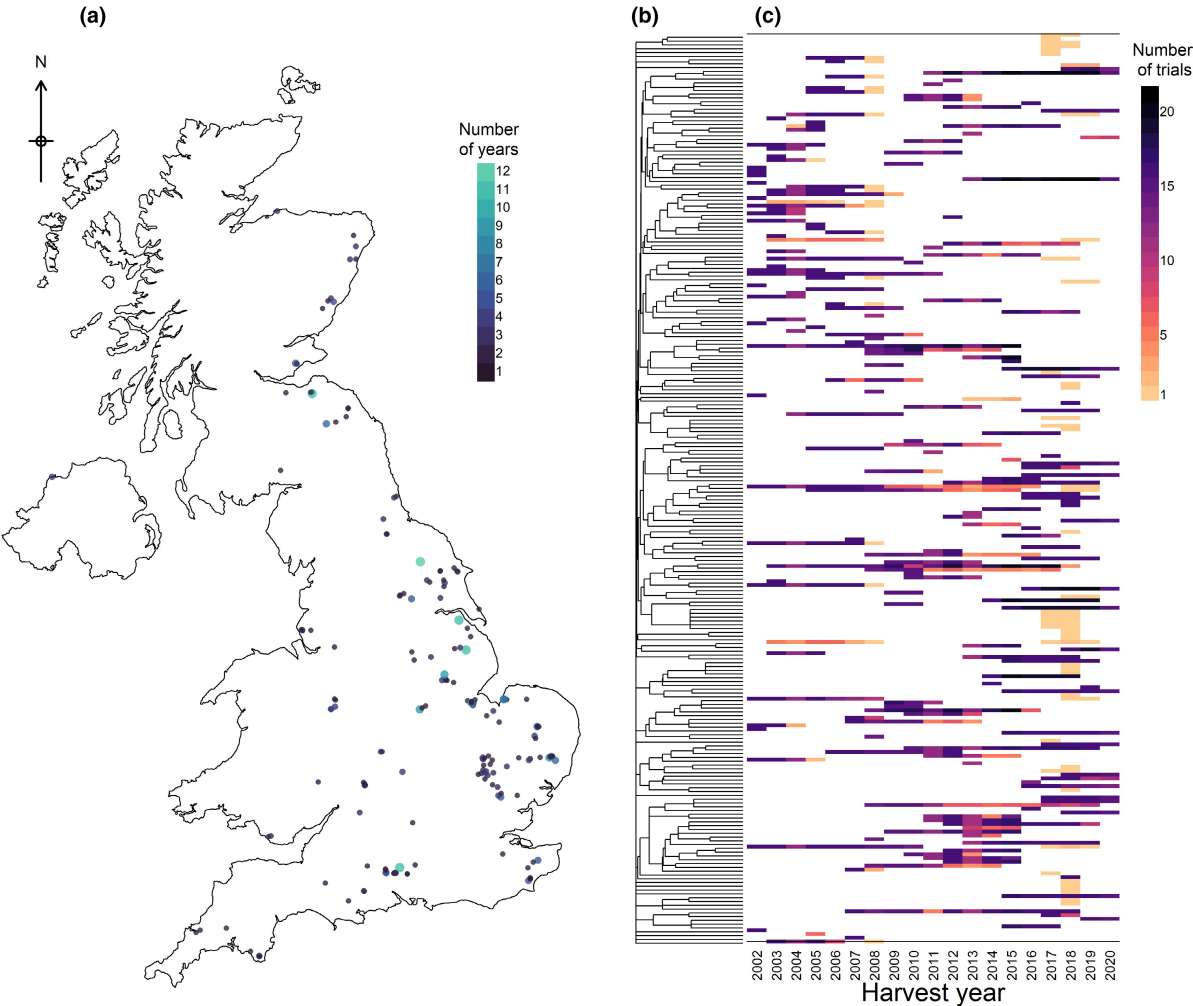
Spatial, temporal and genetic structure of material in the field trials dataset. (a) Locations of the 392 trial environments across the UK. Point size and colour indicate the number of years of trials at that location. Map lines delineate study areas and do not necessarily depict accepted national boundaries. (b) Dendrogram indicating pedigree‐based kinship for the 240 genotypes included and (c) their occurrences in trials each year.

Across the whole dataset, between 15 and 29 (mean = 20.47) trials were included each year, with the exception of 2001 when there were only three trials and only Chopin *W* and Chopin *P*/*L* were recorded for two genotypes. The genotype occurrences per environment were therefore highly unbalanced where each genotype only appeared in trials in a limited number of years (Figure 1a). However, considering the genetic relationships among genotypes, groups of related genotypes were reasonably well balanced across trial years (Figure 1). A total of 11,030, 10,898 and 11,269 observation data points (variety–environment combinations) of protein content, HFN and specific weight, respectively, were included in the dataset while there were 493 and 540 observations of Zeleny sedimentation and Chopin alveograph, respectively.

### 2.2 Envirotyping and enviromic relationship matrix

Characterisation of trial environments (envirotyping) was conducted considering environmental covariables (ECs), including weather information and soil properties, for past trial data and future projections. Soil physical and biochemical properties for each trial location were derived from the *SoilGrids* global resource (De Sousa et al., 2021), available at: https://soilgrids.org/. These included five soil features: soil texture (percentages of sand, clay and silt), soil organic carbon (dg kg^−1^), nitrogen (cg kg^−1^), pH and cation exchange capacity at pH 7 (mmol_c_ kg^−1^). All values were means from the 5‐15 cm depth layer. The weather variables were selected to ensure direct comparison and integration between observed and projected climate datasets and are summarised in Table 2. We consider monthly average ECs to provide relevant temporal resolution to describe crop development, and use monthly average data for the period between typical planting and harvest times for winter wheat in the UK (i.e. October to August). The weather records for each field trial environment were derived from the interpolated 12 km gridded HadUK‐Grid dataset from the years 2001 to 2020 (Hollis et al., 2019; Met Office Hadley Centre, 2018) using the latitude and longitude for each field site. Future projections for the same weather variables were taken from the UKCP18 projections (Lowe et al., 2018) that were based on the Representative Concentration Pathways (RCP) 8.5 high emissions scenario. The envirotyping information was then used to create a *q* environments × *k* environmental covariates matrix of EC values per trial environment. The EC matrix was scaled and mean centred and used to compute a *q* environments × *q* environments enviromic relationship matrix (ERM) which describes the similarities among pairs of field trials across years using a Gaussian kernel. Preliminary analysis investigated performing feature selection using gradient boosting machine learning models (Friedman, 2002) to select the most relevant ECs for each trait but this was not found to increase within‐trial prediction accuracy. This was supported by Costa‐Neto et al. (2022) who also advocated using all ECs in ERM for prediction of new genotypes in new years. Therefore, a non‐linear kernel was calculated using all ECs according to Costa‐Neto, Fritsche‐Neto, et al. (2021) and Costa‐Neto, Galli, et al. (2021):

$$\mathrm{ERM}=\exp\left\{-h\frac{{\left\|w-w^{\prime }\right\|}^{2}}{\theta }\right\},$$

where w is the scaled EC matrix, ${\left\|w-w^{\prime }\right\|}^{2}$ is the Euclidean Distance between each element of the EC matrix (*n* environments × *m* ECs), θ is a scaling factor assumed as the mean value of the Euclidean distance matrix and h is a bandwidth factor which was assumed to be 1 as default. The ERM therefore had dimensions of *n* environments × *n* environments. This approach was implemented in R using similar methods to those detailed in the EnvRtype package (Costa‐Neto, Fritsche‐Neto, et al., 2021; Costa‐Neto, Galli, et al., 2021).

**TABLE 2:** Description of weather variables used to characterise each trial environment

Variable	Description	Units
Rainfall	Average daily precipitation totals over the calendar month	mm day^−1^
Mean temperature	Average of daily mean air temperatures over the calendar month	°C
Min temperature	Average of daily minimum air temperatures over the calendar month	°C
Max temperature	Average of daily maximum air temperatures over the calendar month	°C
Humidity	Average of hourly mean relative humidity over the calendar month	%
Pressure	Average of hourly mean air pressure at sea level over the calendar month	hPa
Wind speed	Average of hourly mean wind speed at 10 m above ground level over the calendar month	m s^−1^

### 2.3 Genotyping and genomic relationship matrix

The genomic relationship matrix (GRM) was computed by combining pedigree and single nucleotide polymorphism (SNP) marker array data to characterise the genetic relationships among genotypes in the study. While marker data offer a more accurate representation of realised identity by state of inherited genetics, ancestry and pedigrees can offer complementary information for average identity by descent of inherited haplotypes and can often be used together in genomic prediction models (Crossa et al., 2010; Velazco et al., 2019).

First, the pedigree‐based kinship matrix (**A** matrix) among all 240 genotypes included in the trials dataset was calculated based on information both from an updated version of pedigree data for released varieties (Fradgley, 2021), as originally described by Fradgley et al. (2019), as well as directly from personal communication with the wheat breeder at DSV UK for breeding lines. The process of computing the **A** matrix from pedigree data was conducted using the ‘kinshp2’ R package (Sinnwell et al., 2014), where six additional generations of inbreeding were included to account for inbred line development. The **A** matrix therefore had dimensions of *n* genotypes × *n* genotypes and kinship values were between zero and one. The diagonal of the **A** matrix all had values close to one describing the kinship between inbred lines and themselves, while sibling lines with the same parent genotypes or parent and offspring relationships had a kinship value close to 0.5. Genotypes with no ancestral pedigree connection were assumed to have a kinship of zero.

Secondly, we calculated the realised genomic kinship matrix (**G** matrix) based on SNP marker data available for 213 varieties in common with the **A** matrix. The majority of these genotype data for released varieties were from the 90 k SNP array (Wang et al., 2014) dataset, as used by Fradgley et al. (2019), while DSV UK breeding lines were genotyped with an in‐house 25 k SNP array. The 10,757 markers that were polymorphic and in common between these two arrays were filtered by removing markers with more that 10% missing data or <0.05 minor allele frequency across genotypes. Remaining missing data were then imputed using the ‘missForest’ R package (Stekhoven & Bühlmann, 2012). To reduce redundancy in the dataset, the remaining 8848 markers were then pruned by removing one of each marker pair with Pearsons's correlation coefficients greater than 0.8, leaving 3275 markers for further analysis. Then, the linear genomic realised additive kinship matrix (**G**) was calculated as described by VanRaden (2008)

$$G=\frac{{\mathrm{MM}}^{\prime }}{p},$$

where M is the scaled *n* individuals × *m* SNP marker matrix and p is the number of SNP markers (3275).

Finally, the **A** and **G** matrices were combined based on Henderson's standard mixed model equations (Henderson, 1976) by scaling **G** to match values in **A**, as described by Pérez‐Rodríguez et al. (2017). This resulted into a combined and weighted **H** matrix as implemented by Velazco et al. (2019) as

$$H=\omega A+\left(1-\omega \right)G_{s},$$

where A is the pedigree relationship matrix, $G_{s}$ is the scaled genomic relationship matrix and ω is a weighting factor between 0 and 1. A value of ω = 0.2 was used that was found to perform well by Velazco et al. (2019) to preferentially weight the **G** matrix. For genotypes with pedigree information but no marker data, unweighted values of **A** were used in the **H** matrix. The resulting *n* × *n* individuals **H** matrix was used as the GRM for subsequent genomic prediction models.

### 2.4 Genetic and environmental correlations

To assess the extent of common genetic and environmental influences between different quality traits, genetic and environmental trait correlations were estimated using available data. However, due to the unbalanced nature of available observations across environments for each trait we fitted pairs of traits (rather than all traits together) for all trait combinations with Bayesian multi‐trait linear mixed models (multi‐response) with unstructured residual covariance using the multi‐trait function from the ‘BGLR’ package in R (Pérez, & de los Campos, 2014; Pérez‐Rodríguez & de los Campos, 2022)

$$Y=1_{n}\mu^{\prime }+g+E+\varepsilon ,$$

where the matrix Y is of size n×t where the columns represent the phenotypic values of each of the *t* traits and the rows are the observations of the cultivars or genotypes. The vector μ is of size *t* × 1 and represents the intercept or mean of each trait and is considered as a fixed effect. The vector of ones $1_{n}$ is of size n. The matrix ***g*** denotes the random genetic effects of order n×t and follows a normal distribution $g∼N_{n\times t}\left(0Z_{g}K_{g}Z_{g}^{\prime },U_{g}\right)$ where $Z_{g}$ is an incidence matrix of the cultivars of order $n\times n_{L}$, (where $n_{L}$ represents the number of different varieties), $K_{g}$ is the relationship matrix of the genotypes of size $n_{L}\times n_{L}$ (in this case the **H** matrix as defined above), and $U_{g}$ is a variance–covariance matrix of genetic effects between the traits of size *t* × *t*. The matrix ***E*** denotes the random environmental effects of order n×t and follows a normal distribution $E∼N_{n\times t}\left(0,Z_{E}K_{E}Z_{E}^{\prime },U_{E}\right)$ where $Z_{E}$ is an incidence matrix for trial environments of order *n* × *n*_*E*_ ($n_{E}$ represents the number of environments), $K_{E}$ is the relationship matrix of the environments of size $n_{E}\times n_{E}$ (in this case is an identity matrix) and $U_{E}$ is a variance–covariance matrix of environmental effects between the traits. Random errors are represented by the matrix ***ε*** of order n×t that follows a normal distribution $\varepsilon ∼N_{n\times t}\left(0,I,\sum_{t}\right)$ where the identity matrix I is of dimension $n_{T}\times n_{T}$. Multi‐trait models were run with 10,000 iterations of Markov Chain Monte Carlo (MCMC) to optimise prior distributions, of which 5000 were burn‐in, and a thinning value of 2.

### 2.5 Prediction models

This study considers models involving GRM and ERM for the phenotypic prediction across years. Here we used Bayesian Linear Regression with Reproducing Kernel Hilbert Spaces (RKHS) (Gianola & van Kaam, 2008). We compared four prediction models for each trait, where the first two included only genetic information from the GRM, while the second two additionally included the ERM and the G × E interaction. First, the separate kernels that model each of the different genetic, environmental and G × E components were constructed according to the trial design matrices for each trait as described by Costa‐Neto, Fritsche‐Neto, et al. (2021) and Costa‐Neto, Galli, et al. (2021). Then, Bayesian Hierarchical models were fit using these kernels with the BGGE function of the ‘BGGE’ R package (Granato et al., 2018). All models were run with 10,000 iterations of Markov Chain Monte Carlo (MCMC) to optimise prior distributions, of which 2000 were burn‐in, and a thinning value of 2. Below we detail each one of the four model structures and its assumptions in terms of effects and kinships.

The baseline model of this study considers only the main genetic effects (hereafter named: MM) (Lopez‐Cruz et al., 2015). This model can be described as

y=μ1+E+g+ε,

where ***y*** is the vector of the phenotypic records from the field trials (*n* × 1 dimension), μ1 is the fixed‐effect overall mean (*n* × 1 dimension), E is the random environmental effect, assumed as $E\sim N(0,K_{E}\;\sigma_{E}^{2}$) where $\sigma_{E}^{2}$ is the variance of E and $K_{E}$ is the *n* × *n* dimension kernel for environmental variation, computed as: $K_{E}=Z_{E}Z_{E}^{\prime }$. matrix $Z_{E}$ is the incidence matrix for the environments in the dataset. The vector of random genetic effects were assumed as $g∼N\left(0,K_{g}\sigma_{g}^{2}\right)$, where $\sigma_{g}^{2}$ is the genetic variance and $K_{g}$ is the *n* × *n* dimension kernel for genetic effects, computed as: $K_{g}=Z_{g}HZ_{g}^{\prime }$, in which $Z_{g}$ is the incidence matrix for the individuals, and H is the genomic relationship matrix (GRM) as described above. Finally, for this model, we assume an independent structure for the residuals, as $\varepsilon ∼N\left(0,\sigma_{\varepsilon }^{2}I\;\right)$ where $\sigma_{\varepsilon }^{2}$ is the error variance.

Further to the MM model, we added a random G × E interaction term, which resulted in the second model of this study, the multi‐environment, single variance G × E deviation model (MDs). According to Lopez‐Cruz et al. (2015), this model is described as

y=μ1+E+g+gE+ε,

where the random effect gE for G × E interactions is included, where $\mathrm{gE}∼N\left(0,\left[K_{g}⊙K_{E}\right]\sigma_{\mathrm{gE}}^{2}\right)$ with ⊙ indicating the Haddamard product and $\sigma_{\mathrm{gE}}^{2}$ the variance of gE.

We then extend both the MM and MDs models to include predictable G × E interaction terms between the genomic and enviromic kernels (e.g. soil and weather variates), resulting in kernel‐based reaction‐norm models that include genotype specific response over an environmental gradient. The environmental variation considers a nonlinear enviromic‐aided ERM (Ω), resulting in the updated enviromic‐aided environmental kernel, hereafter named as $K_{W}=Z_{E}\Omega Z_{E}^{\prime }$. Consequently, the addition of a kernel‐based reaction‐norm structure (gW) is given by: $\mathrm{gW}∼N\left(0,\left[K_{g}⊙K_{W}\right]\sigma_{\mathrm{gW}}^{2}\;\right).$ Thus, this reaction‐norm extension of the MM (here after named as RNMM) can be described as

y=μ1+E+g+gW+ε.

Similarly, to the extension of the MM to the RNMM model, the reaction‐norm extension of the MDs model (hereafter named as RNMDs) considers a G × E deviation (gE) and a reaction‐norm structure (gW), which can be described as

y=μ1+E+g+gE+gW+ε

with $\mathrm{gW}∼N\left(0,\left[K_{g}⊙K_{W}\right]\sigma_{\mathrm{gW}}^{2}\right)$ and $\mathrm{gE}∼N\left(0,\left[K_{g}⊙K_{E}\right]\sigma_{\mathrm{gE}}^{2}\right)$.

Therefore, both MM and MDs can only model genetic and unpredictable random G × E effects in the observed set of environments and will make the same predictions of genotype performance in new untested environments. On the other hand, RNMM and RNMDs models can make use of EC data that characterises conditions in both observed and new environments and will make more specific predictions of genotype performance in each new environment according to predictable G × E.

### 2.6 Model quality assessment

The unbalanced nature of the trials dataset where limited numbers of genotypes were tested in each trial year presents challenges by confounding genetic and environmental effects and risks overfitting and exaggerating G × E in predictions from the models. Modelling the genetic effects as a continuous GRM kernel space rather than considering genotypes as independent factors helps avoid this overfitting. To assess the accuracy and potential for predictions to be made in future years, a ‘leave‐one‐year‐out’ cross‐validation strategy for untested genotypes in untested years was applied to replicate a realistic situation faced by breeders wishing to predict the performance of lines that are genotyped but have not been tested in any environments. For this, cross‐validation was performed so that data from each year was removed in turn and predicted by models that were trained on all the remaining years. In addition, genotypes that occurred in common between test and training folds were removed from each training fold so that only predictions of untested genotypes were assessed. The predictive accuracy of overall year‐to‐year environmental effects on quality traits in response to climate variability was assessed as the Pearson's correlation coefficient between the mean values of observed and out‐of‐fold predicted trait values for each year. Genomic predictive accuracy of quality traits was also calculated for each trial environment as the Pearson's correlation between the observed trait data and out‐of‐fold predictions. Mean and median prediction accuracies across all trial environments were compared across prediction models and statistical significance between means was tested using paired t‐tests between both MM vs RNMM and MDs vs RNMDs models. After model cross‐validation, full models were fitted for each trait and prediction model using all data from all trial years. Finally, estimates of variance components for genetic, environmental and G × E effects and residual unexplained variance were derived from these full models for each trait.

### 2.7 Projection of future climate effects

A major goal of this study was to understand the phenotypic landscapes of each quality trait in the context of climate change. For this, RNMM models were fitted including all past observed data (past phenotypic and environmental information). The models were then used to predict phenotypes for 20 randomly selected wheat variety genotypes under both the recently observed climate and projected future climate conditions in all 1707 of the 12 km gridded square locations across the UK used in the HadUK‐Grid and UKCP18 datasets. Therefore, a total of 68,280 new genotype–environment combinations were predicted for each trait. The recently observed climatological average was derived by averaging monthly weather covariates over the years 2000 to 2020 in the HadUK‐Grid weather dataset. Similarly, the future climatological average was derived by averaging the projected monthly weather covariates over the years 2050 to 2069, as well as across all 12 individual ensemble members in the UKCP18 weather dataset. Prior to averaging across ensemble members, the UKCP18 projections were bias‐corrected, in which individual model biases in the mean and variance of each variable were corrected to match those observed in the HadUK‐Grid weather dataset using the following formula

$$X_{\mathrm{fut}-\mathrm{bc}}\left(t\right)=\bar{O_{B}}-\bar{X_{B-\mathrm{raw}}}+\bar{X_{\mathrm{fut}-\mathrm{raw}}}+\frac{\sigma_{\mathrm{OB}}}{\sigma_{\mathrm{XB}-\mathrm{raw}}}\left(X_{\mathrm{fut}-\mathrm{raw}}\left(t\right)-\bar{X_{\mathrm{fut}-\mathrm{raw}}}\right),$$

where X = UKCP18 model outputs; O = HadUK‐Grid observations; B = Baseline period (2000–2019); fut = Future period (2050–2069); raw = unadjusted; bc = bias corrected; $\left(t\right)$ = climate data time series; σ = standard deviation of climate data. Overbar indicates average. A transform was applied to rainfall (natural logarithm of rainfall) and relative humidity ($\sqrt{100-\text{relative humidity}}$) prior to bias‐correction, to ensure the bias‐corrected values remained within physical bounds. All 12 km grid boxes and weather covariates were therefore comparable between the observed average and future climate environments. For soil data extracted from the SoilGrids global resource (Poggio et al., 2021), data for each soil property variable for each 12 km grid box that was missing due to proximity to urban land use were imputed with the median value.

### 2.8 Prediction of trait stability across projected future year‐to‐year environmental variation

To further test to what degree the predictable G × E in prediction models could be exploited to mitigate year‐to‐year climate variability impacts on UK wheat quality traits, we also fitted models using all 240 available genotypes and the full observed dataset to predict their performance under a wide range of climatic conditions by sampling years from each individual UKCP18 ensemble member. In contrast to using the ensemble mean as above, this represents physically plausible year‐to‐year climate variability, under the RCP8.5 emissions pathway. By sampling each of the 20 years between 2050 and 2069 and across all 12 ensemble members, we obtained 240 independent year environments in which to assess the genotypes' performance. We sampled this climatic variability at three locations in the East, North and South‐west of the UK which were projected to experience contrasting climate change impacts so that a total of 172,800 new genotype–environment combinations were predicted for each trait. In summary, these represent a wide range of 720 simulated environments that are self‐consistent throughout the year, with realistic temporal variation around the expected future average climate of 2050 to 2069, and that are independent from the years observed in the trial dataset.

Variation in genotypic sensitivity to the expected future temporal environmental variation was assessed on the predicted 172,800 genotype–environment combinations using joint regression stability analysis as first described by Yates and Cochran (1938) and further outlined by Finlay and Wilkinson (1963) where the performance of each genotype is regressed linearly against the mean performance of all genotypes in each environment as an index of environmental quality. This analysis was possible for the completely balanced projected dataset rather than the highly unbalanced observed historical trial data. The height of the fitted line corresponds to the overall genetic value of each genotype while the slope (*b*) was used as the stability index of genotypic sensitivity to temporal environmental variation due to the G × E variance component of prediction models. An index of risk was also defined as the percentage of the 240 projected year environments in which the predicted trait value for each genotype did not meet the thresholds as defined by the UK variety quality criteria for bread wheat export (AHDB, 2022a). These were grain protein content between 11 and 13%, HFN over 250 s, specific weight over 76 kg hl^−1^, Chopin *W* above 170 and Chopin *P*/*L* below 0.9. An arbitrary threshold of 30 was used for Zeleny sedimentation volume.

### 2.9 Assessment of specific weather variable effects on quality traits

Because the observed multi‐environment trial dataset is highly structured across years, determining specific relationships between quality traits and specific weather variables was problematic due to strong confounding effects of genotype and geographic location. We therefore used both a machine learning approach and correlation analysis to quantify weather variable effect importance and direction of effect on the predicted trait values from G × E prediction models including all 240 genotypes predicted in all 240 projected year environments. This reveals some of the specific environmental effects captured in the ERM Gaussian kernel and modelled in the Bayesian linear regression models. For each trait, the ECs were used as features in random forest regression ensemble learning prediction models to predict the mean trait value in each projected environment across all genotypes using the ‘randomForest’ package in R. Default parameters were used where one third of the feature variables were randomly sampled as candidates at each split, the minimum size of terminal leaf nodes was five, and 500 trees were grown for each random forest model. Relative importance values of ECs were then derived from these full models as the mean decrease in mean squared error across all trees. The direction of variable effects was also determined from pairwise correlation coefficients between each EC and the mean trait value in each projected environment.

## 3 RESULTS

### 3.1 Environmental covariates enable prediction of G × E

All combinations of traits were found to have positive genetic correlations suggesting some degree of positive pleiotropic genetic control. However, both positive and negative environmental correlations were found (Figure S1). Protein content, Zeleny and Chopin *W* all had strong positive environmental correlations, whereas HFN and specific weight both had negative environmental correlations with the gluten quality traits (Zeleny, and both Chopin *W* and *P*/*L*).

**FIGURE 2:**
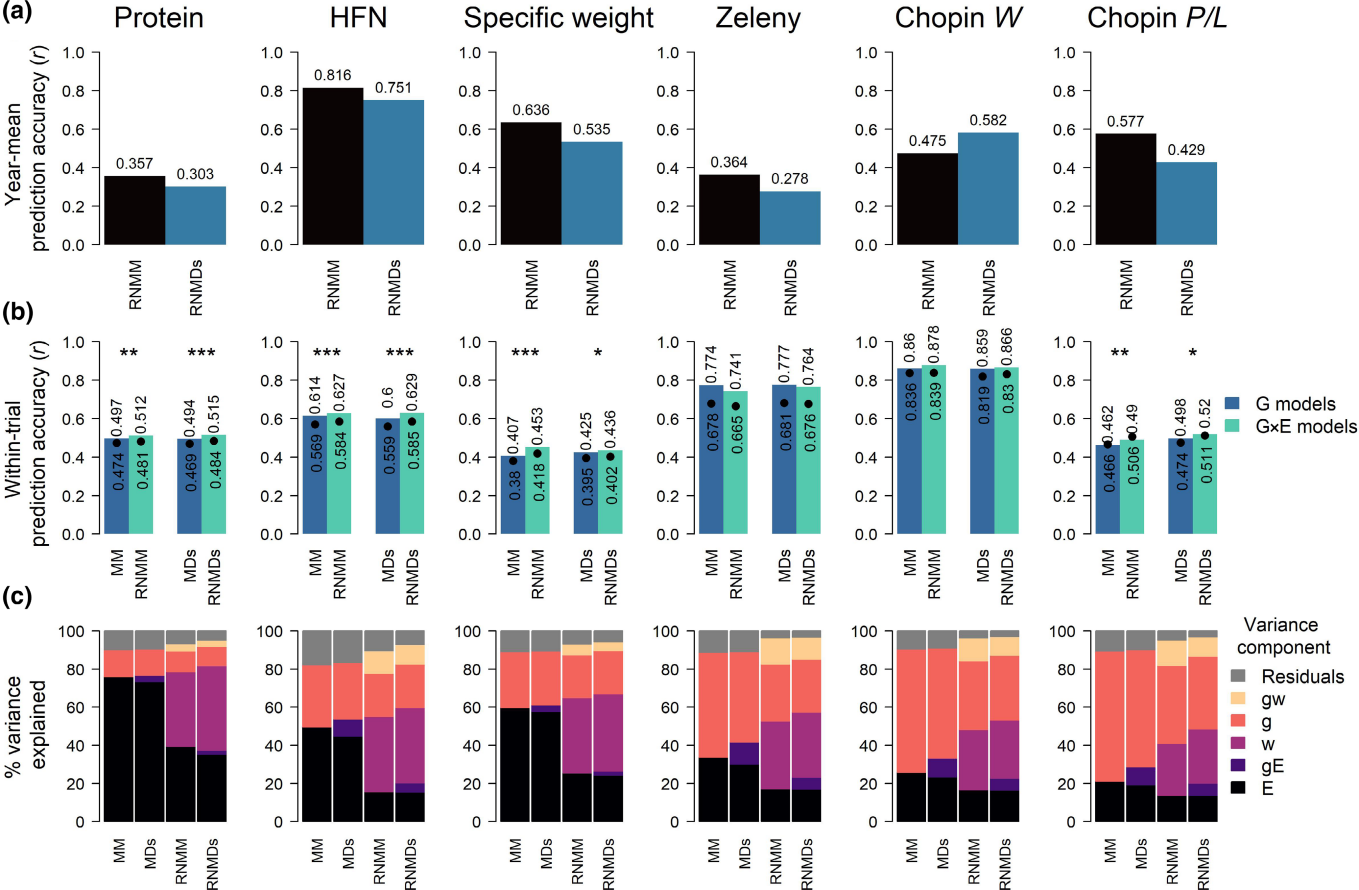
Models including environmental covariate improve the prediction accuracy for wheat quality traits. (a) Comparison of prediction accuracy (correlation between observed and predicted data) of mean trait values for each trial year for both reaction norm models (RNMM and RNMDs) that incorporate environmental covariates into models for each quality trait. HFN, Hagberg Falling number. (b) Comparison of cross‐validated within‐trial genomic prediction accuracy between genetic models (MM and MDs) that use only genetic data and equivalent reaction norm models (RNMM and RNMDs) for untested genotypes in untested years cross‐validation. Bars indicate median while black points indicate the mean prediction accuracy across all trial environments. Median and mean values are also shown above and below bars and points respectively. Statistical significance level between means from paired *t*‐tests are shown above each pair of G and G × E prediction model comparisons where *p < .05, **p < .01, and ***p < .001. (c) Percentages of the phenotypic variation explained by each variance component for each prediction model for all traits. Variance components are described in the methods where E indicates the random effect of environment, gE indicates the interaction between genotype and the random environmental effect, w indicates the predictable environmental effect that can be explained by weather and soil environmental covariates, g indicates the genetic effect estimated from markers and gw indicates the predictable interaction between g and w.

Prediction accuracy (correlations between predicted and observed values in the historical trials) for year‐mean (across locations and genotypes) trait values for HFN and specific weight (Figure 2a) varied between 0.53 and 0.82. These moderate to high prediction accuracies suggest that both RNMM and RNMDs reaction norm G × E models were largely able to predict overall environmental effects of year‐to‐year climate variation within the dataset (Figure 2a). Grain protein content had a large environmental variance component, but lower year‐mean prediction accuracies (0.3–0.36) suggesting that environmental variation affecting this trait was more influenced by geographic location and soil fertility than by year‐to‐year weather variability. For all quality traits, except Zeleny and Chopin *W*, a small but statistically significant increase in mean within‐trial prediction accuracies of genotype performance was found with the inclusion of environmental covariates (RNMM and RNMDs models) compared to models that included only genetic data (MM and MDs models) (Figure 2b).

With models fitted over the whole dataset of observed historical trials, only a relatively small proportion of the phenotypic variance was explained by predictable G × E variance components for grain protein content (3.95% and 3.18% for RNMM and RNMDs models, respectively; Figure 2c). In comparison, other traits had larger genetic G × E variance components. For HFN, this was 11.91% and 10.26% for RNMM and RNMDs models, respectively. The environmental component of HFN, which explained approximately half of the overall phenotypic variance, could mostly be predicted by including weather and soil covariates in RNMM and RNMDs models (Figure 2c). Gluten quality traits (Zeleny sedimentation, Chopin *W* and Chopin *P*/*L*) had particularly large genetic and G × E variance components, which also reflected the generally high prediction accuracy for these traits (Figure 2b). For all traits, the addition of environmental covariates in reaction norm models (RNMM and RNMDs) successfully reduced the estimated unexplained residual variance component. For some traits related to gluten quality residual variance was reduced from between 8.9% and 11.23% to between 3.0% and 4.7% (55.5% to 70.0% reduction), while for other traits (grain protein content, HFN and specific weight) it was reduced from between 9.9% and 17.8% to between 6.6 and 10.4% (32.7% to 41.6% reduction) for RNMM models and from between 9.6% and 16.6% to between 4.7% to 7.2% (48.4 to 56.7 reduction) for RNMDs (Figure 2c). These results demonstrate successful prediction of G × E and the small remaining residual variance components suggest that even models that could perfectly predict the G × E component would have only slightly increased accuracy and importantly, that predictions from these models have realistic G × E variation.

### 3.2 Predicted effects of climate change on wheat quality traits are different across UK regions

**FIGURE 3:**
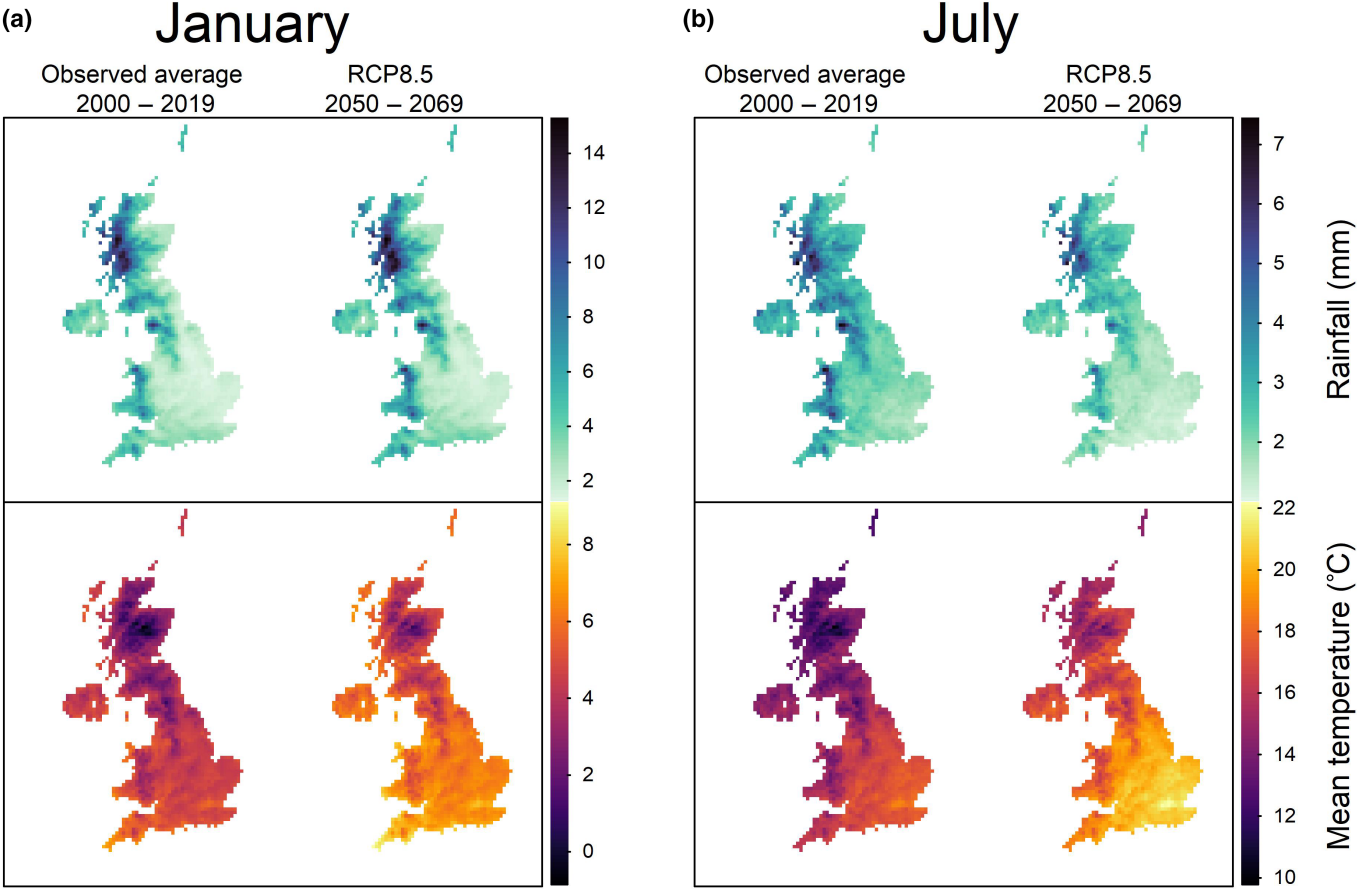
Overview of projected changes in UK summer and winter climate. Comparison of climatological average rainfall and temperature patterns in (a) January, and (b) July across the UK at 12 km grid square resolution between the observed average climate between 2000 and 2019 (derived from the HadUK‐grid weather dataset) and projected future climate conditions between 2050 and 2069 (derived from the bias corrected UKCP18 climate projections dataset and averaged across all 12 ensemble members). Map lines delineate study areas and do not necessarily depict accepted national boundaries.

Genomic RNMM prediction models effectively explained a significant proportion of G × E variance components, so we used these models to predict the overall impacts of climate change on UK wheat quality traits based on UKCP18 climate projections. We compared observed weather data from 2000 to 2019 with the RCP8.5 future climate conditions of 2050 to 2069. Figure 3 illustrates and exemplifies the key contrasting summer and winter trends in projected climate change at all locations in the UK. While there is significant year‐to‐year and within‐year variability, these projections show warmer and wetter winters on average, with this effect being most pronounced over elevated regions in the western UK (Figure 3a and Figure S2), and hotter and drier summers on average (Figure 3b).

**FIGURE 4:**
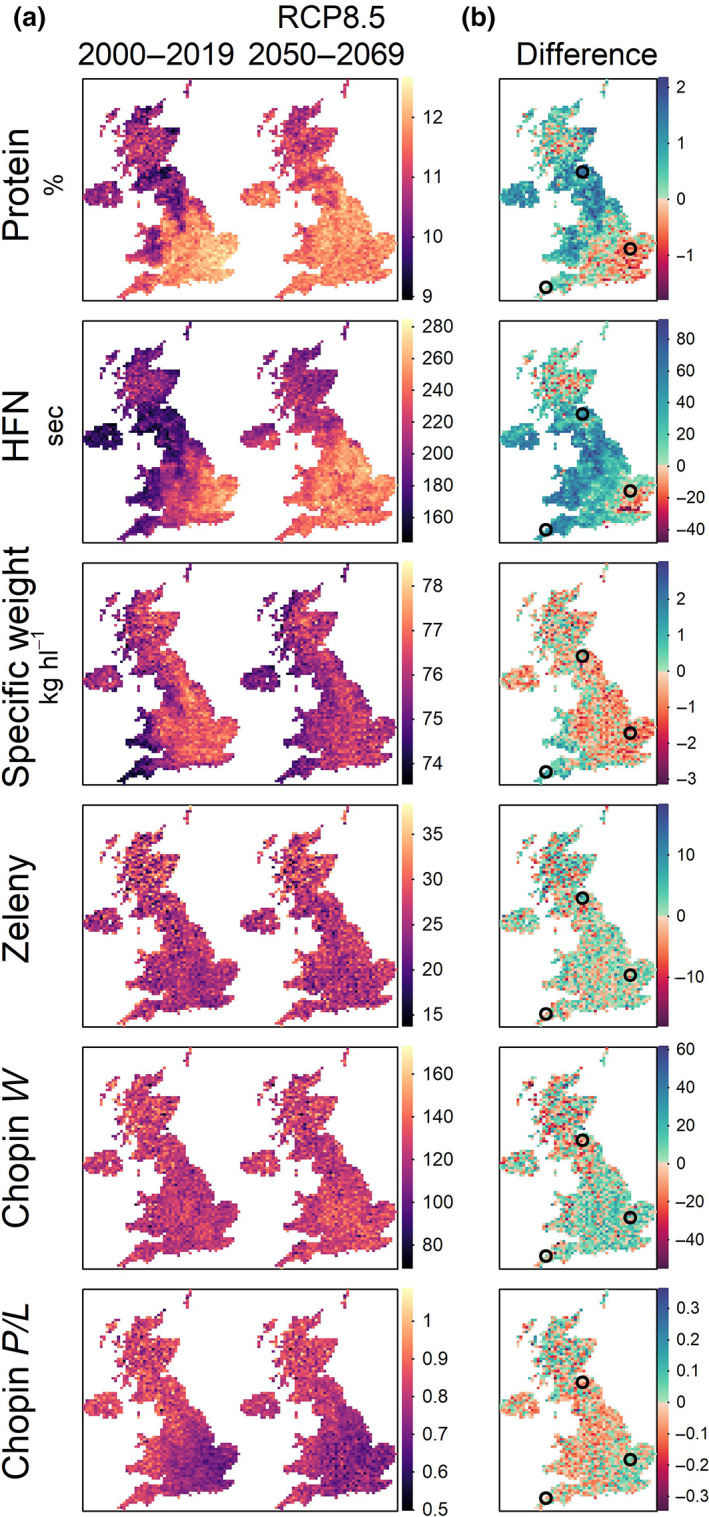
Changes for quality trait values due to projected climate change under RCP8.5. (a) Heatmaps of quality trait values at all 12 km grid square locations around the UK for the observed average climate (2000–2019), compared to the projected future climate conditions (2050–2069), and (b) for the differences between them. Three circles indicate locations where likely effects of year‐to‐year variation were also predicted (see Figure 5). HFN, Hagberg falling number. Map lines delineate study areas and do not necessarily depict accepted national boundaries.

For each of the 12 km grid boxes covering the UK land area (*n* = 1707), we predicted the quality traits for a representative subset of 20 wheat genotypes at both the observed average (2000–2019) and projected future climate conditions (2050–2069) so that 68,280 simulated genotype–environment combinations were modelled. On average, positive effects of future projected climates were predicted for grain protein content and HFN, with negative effects for specific weight (Figure 4a). Differences in Zeleny sedimentation and Chopin alveograph measurements for gluten quality were predicted to be neutral (Figure 4a). However, these effects were dependant on geographic location around the UK. Under the observed average climate of 2000–2019 the East of England was predicted to produce the highest wheat quality in terms of protein content and HFN, accurately reflecting the current distribution of milling wheat in the UK (Figure 4b). However, decreases in both protein content (by more than one percentage point), and HFN (by as much as 40 s), were predicted in the East under this future climate scenario. Regions in the North and South‐west of the UK, where these traits are generally lower in current climates, were predicted to become more favourable, with grain protein content predicted to increase by as much as two protein percentage points and 80 s for HFN (Figure 4b). Specific weight was predicted to decrease in most regions, but particularly where it is highest in the current climate (East of England). This decrease could be as much as 3 kg hl^−1^ (East of England), but a few locations in the South‐west and Wales were predicted to increase by over 2 kg hl^−1^ (Figure 4). These trends also explain the reduction in variance in predicted trait values across geographical regions for both protein content and specific weight (Figure 4a). Climate change effects were much smaller for other gluten quality traits (Zeleny and Chopin alveograph traits) that had smaller environmental variance components (Figure 2).

In this RCP8.5 climate average scenario, the percentage of the UK land area that may be suitable for high quality wheat growing, as assessed by an average protein content above a threshold of 12.5%, across the 20 genotypes in this analysis was found to decrease from 0.5% to 0.06%. Furthermore, the area with average specific weight above 76 kg hl^−1^ decreased from 57.4% to 37.0%, while the area with average HFN above 250 s increased from 4.9% to 17.9%.

### 3.3 Associated weather covariables explain contrasting climate change effects across UK regions

**FIGURE 5:**
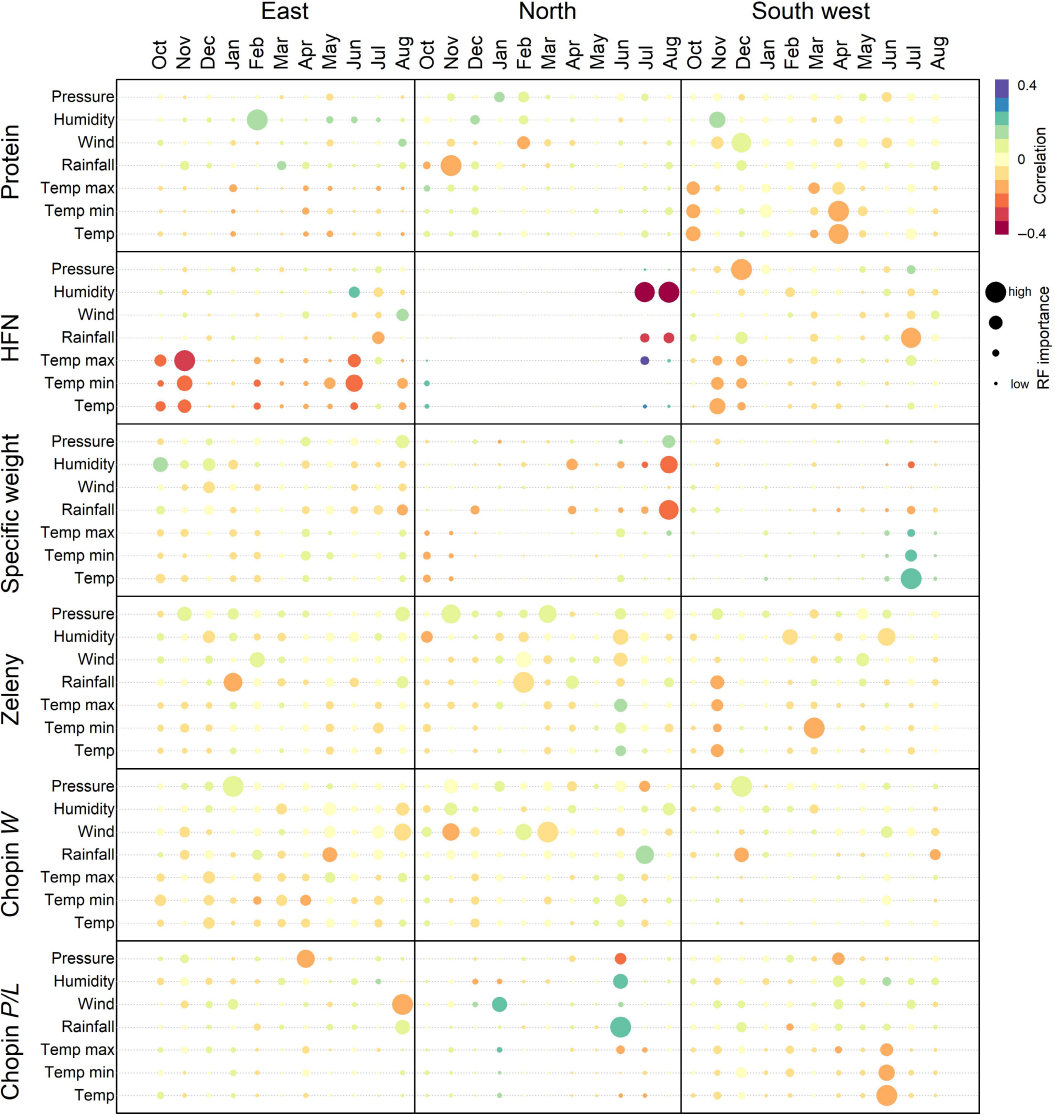
Correlation and relative importance of specific weather covariables on mean quality trait predictions across all genotypes at three locations in the UK in future projected year‐to‐year simulated environments. Weather covariables were those used to construct the environmental relationship matrices in predictions model. Locations were selected based on their contrasting projected climate change effects (Figure 4). HFN, Hagberg falling number. Data are shown as correlation and the scaled importance scores derived from random forest models fitted on the trait values predicted from RNMM models (Figure 2).

To investigate the effects of future year‐to‐year weather variation in more detail, we selected three contrasting locations and predicted the performance of all 240 wheat genotypes. Locations in the East, North and South‐west of the UK (shown in Figure 4b) were selected based on contrasting expected climate change effects. We predicted the wheat quality traits in the 240 simulated year‐environments derived from the 12 different climate projection ensemble members for 20 years between 2050 and 2069 in each of the three UK regions so that 172,800 simulated genotype–environment combinations were predicted. We used random forest ensemble prediction models and correlation analysis to determine the importance and effect direction of specific environmental covariates on mean predicted trait values per projected year‐environment. This approach revealed specific weather trends driving year‐to‐year environmental effects in the otherwise hard‐to‐interpret models that were effective at predicting G × E (Figure 5).

Contrasting weather effects for each trait among the three locations reflected their different projected future climate conditions. For example, in the North and South‐west, where projected climate change had a positive effect on HFN, there was a clear negative association between HFN and both relative humidity and rainfall before harvest in July which were both projected to decrease under future climate scenarios. By contrast, the negative effect of projected climate change on HFN in the East could be explained by the greater negative association with temperature throughout the growing season, and a smaller negative association with rainfall in July (Figure 5). For specific weight, climate projections on average show increased rainfall over winter months up until April. Together with the weak but negative association between rainfall in April and specific weight at the locations in the North and East, this may explain the generally negative projected climate change effects for this trait (Figure 5). On the other hand, the large positive association between specific weight and temperature from June to August at the South‐west location may explain the positive climate change effect at this location (Figure 4). Both Chopin alveograph *W* and *P*/*L* traits were less affected by the environment but in general, both high temperature and low rainfall late in the growing season between June and August were negatively associated with Chopin *P*/*L* values both in the North and South‐west locations. This also reflects favourable climate change effects because low *P*/*L* values are good for bread baking quality (AHDB, 2022a).

### 3.4 Predictions of G × E responses suggest little potential for breeding to mitigate effects of year‐to‐year environmental variability

**FIGURE 6:**
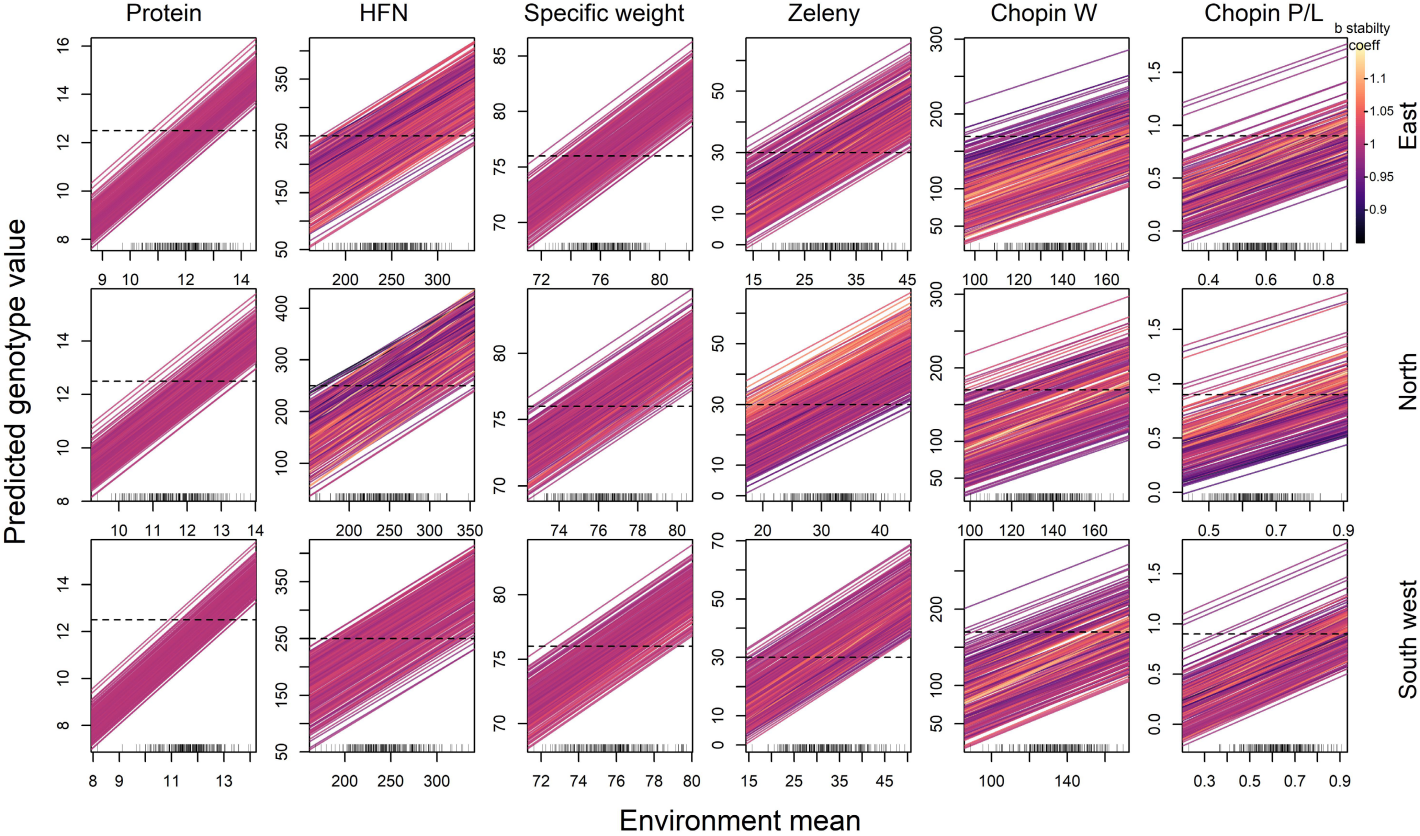
Finlay–Wilkinson joint regression stability analysis for 240 wheat genotypes over 240 future RCP8.5 projected year environments at three locations. For all quality traits, lines represent the adaptive response for a specific genotype plotted against the environmental quality gradient defined by the mean trait value across all genotypes in each year environment. Line colour is proportional to slope of the line (*b* stability coefficient). Dashes inside the *x*‐axes indicate the mean trait value of all genotypes in each of 240 projected year environments. Horizontal dashed lines indicate the minimum quality criteria thresholds for each trait.

We predicted the G × E responses of a large number of genotypes across a large number of projected year‐to‐year environmental variation scenarios in order to assess whether wheat breeding can exploit the predictable G × E to mitigate adverse future climate effects. Genotype performances were modelled at three locations in 240 simulated year scenarios derived from the 12 climate projections from the years 2050 to 2069 in the UKCP18 dataset. Finlay–Wilkinson joint regression analysis was used to assess the performance of each genotype regressed linearly against the mean performance of all genotypes in each year environment at each location (Figure 6). Reflecting the relative size of the overall genetic and G × E variance components across different traits (Figure 2c), this stability analysis revealed very little variation in the slope stability parameter (*b*) that would enable selection to mitigate environmental effects for most traits (Figure S3; Figure 6). This was particularly so for protein content (min = 0.98, max = 1.02 at any of the three locations). Based on our models, only a benefit of 0.05 protein percentage points (1.6% of the environmental effect) would be mitigated by selecting the most stable genotype for the worst‐case simulated year with the most negative projected environmental effects expected in 240 years. However, there did seem to be potential to mitigate environmental effects for HFN. The slope stability parameter had greater variation, particularly at the location in the North (min = 0.83, max = 1.16), where our models suggest that HFN would benefit the most from reduced rainfall before harvest due to climate change. In the most severe year environment for the South‐west, North and East locations, 12.15, 13.06 and 19.45 s, respectively, of HFN (16.0%, 16.5% and 23.2% of the environmental effects, respectively) could be mitigated. There was also some potential for mitigation of year‐to‐year environmental effects for protein quality traits where between 11.7% and 21.8% of the environmental effect could be mitigated for Zeleny sedimentation but these traits had much smaller overall environmental effects.

Comparison of *b* stability coefficients found only weak relationships among traits at the same locations, and for the same trait at different locations (Table S1). Correlations between *b* stability coefficients among traits at the same location were all <.14, except for the closely related Chopin *W* and *P*/*L* dough rheology traits where Pearson correlations were between .25 and .41 across all three locations. Correlations in *b* stability coefficients for the same trait in different locations were higher but still not very strong. HFN and Chopin alveograph traits all had correlations in *b* stability coefficients among sites between .53 and .75, whereas protein content, specific weight and Zeleny sedimentation all had correlations below .3. These low correlations reflect the environmental correlations among traits (Figure S1), and the contrasting climate change effects and patterns of weather variable importance scores among the three locations (Figures 4 and 5). These results suggests that while selecting genotypes with high temporal stability for traits such as protein and specific weight would generally be difficult, selecting a single variety that is stable for multiple quality traits at any one location or has high temporal stability in different regions of the UK would be even more difficult.

We also determined a risk score for each genotype for each trait as the percentage of the 240 projected year‐environments in which the predicted trait value failed to meet the milling quality threshold for each trait. In general, the risk scores were much more related to the overall genetic effect influencing the mean trait value, regardless of the environment, than the *b* stability coefficient (Figure S4). This result suggests much greater potential to reduce risk of poor wheat quality outcomes by exploiting the overall genetic variance component, which is larger and more predictable than the G × E components for these traits (Figure 2c).

Taylor's power law describes how the variance of a trait increases logarithmically with mean trait value (Döring et al., 2015) and has implications for directions of selection in plant breeding programmes. However, the relationships between mean modelled quality trait values and *b* stability coefficients across environments varied considerably across traits and locations. This association was negative for specific weight at two of the three locations (correlation coefficients between −.13 and −.31) indicating that genotypes with higher average specific weight were generally also more stable (low *b* stability coefficients). This was also the case for HFN in two out of the three locations, suggesting that the value of high HFN or specific weight genotypes is greater in more challenging environments. In contrast, mean protein content was positively correlated with *b* stability coefficients at two of the three locations and Zeleny sedimentation at one location. This suggests that positive selection for these two traits may also select for reduced stability and climate resilience.

## 4 DISCUSSION

Given the relevance of wheat in ensuring food security, it is important to quantify the potential threat to wheat grain quality from climate change and assess mitigating strategies. In this study, we aimed to determine the overall effects of projected climate change on UK wheat quality, and to quantify the potential for breeding to mitigate adverse effects of year‐to‐year environmental variation by exploiting predictable G × E. We combined multi‐site and ‐year wheat datasets with large‐scale environmental characterisation (enviromics) and genomic‐enabled prediction models to predict G × E. We explored the impacts of projected climate change on wheat quality in the UK for the period 2050–2069 under a high emissions pathway, RCP8.5. Our results showed positive effects of climate change on key quality traits in some regions that are not currently suited to growing high quality wheat. This represents an opportunity for expanding high quality wheat production, potentially mitigating negative impacts in the current primary high‐quality wheat growing regions in the East of the UK. We found limited potential for wheat breeding to exploit predictable G × E and develop varieties that are resilient to projected year‐to‐year climate variability for most traits, with the exception of HFN. Moreover, different regions of the UK will experience contrasting environmental challenges to quality traits so that it will be particularly difficult to breed varieties that are resilient across the UK for multiple quality traits. The low correlations between modelled genotypic stability at contrasting regional locations also suggests the potential and the need for breeding programmes to select for local rather than broad adaptation.

Inclusion of environmental covariables in genomic prediction models resulted in modest but statistically significant increases in prediction accuracies, which is similar to other studies for wheat yield (de los Campos et al., 2020; Heslot et al., 2014; Jarquín et al., 2014; Ly et al., 2018) or protein content (He et al., 2019). Here we used a more robust cross validation strategy to predict the performance of untested genotypes in untested years rather than year – location combinations, and also found that the prediction accuracies increased when including environmental covariates (Figure 2). Generally high prediction accuracies (>0.4) were found here considering the smaller number of varieties tested in the dataset (particularly for Zeleny sedimentation and Chopin alveograph traits) compared to the above studies, which may be due to the value of the large number of testing environments and the greater genetic, rather than environmental, variance components for these gluten quality traits. The improved performance in robust cross validation testing established that models used here are well suited to the highly unbalanced structure inherent in long‐term variety testing trials which risks confounding between genetic and temporal environmental effects and validates predictions made into future climate scenarios. This study demonstrates the usefulness of integrating existing historical trials datasets across wider geographical ranges and diversities of genotypes from research and applied breeding programmes.

Relationships among environments were modelled using Gaussian kernels where genetic response to the environmental variation and G × E is assumed to follow a multivariate normal distribution (Costa‐Neto & Fritsche‐Neto, 2021). This approach therefore realistically models contrasting environmental effects that can either increase or decrease the realised trait on either side of the optimum environment at the peak of the Gaussian distribution. We show that these differential climate change effects across regions are associated with contrasting weather effects, which supports evidence for a geographical shift of the current best high‐quality wheat growing regions to mitigate projected climate change impacts.

In line with the strong genetic and environmental trade‐off between grain yield and protein content (Galani et al., 2022; Scott et al., 2021), our results suggest a negative effect of increased temperatures throughout the growing season on protein content in the major wheat growing regions in the East of the UK. Indeed, our results showed that the land area with sufficiently high protein levels is likely to decrease to less than one eighth of the area in the current climate average. This also suggests contrasting effects of projected climate change on yield and protein from crop growth models (Asseng et al., 2019) and suggests that opposing positive effects on yield may be found. Rising atmospheric CO_2_ concentrations are also thought to have negative impacts on wheat grain protein content due to the dilution effect of increased yields (Wang & Liu, 2021): however, we could not account for these effects within the observed field trial dataset in the present study as there is unlikely to be variations in CO_2_ concentrations between the UK trial sites that exceeds the long‐term trend. CO_2_ concentrations at Mauna Loa, reflecting global trends, increased linearly by approximately 38 ppm over the period of observed field trials here and so will likely increase by an additional 112 ppm by the projected future period in 50 years (Keeling et al., 2005) while the spatial variation in CO_2_ concentrations among trial environments in the UK is likely marginal compared to the temporal trend.

HFN is well known to be negatively impacted by low temperatures and rainfall inducing pre‐harvest sprouting when the grain is fully mature (Mares, 1993), but more complex negative effects of high temperature at earlier stages of grain development are also known to impact HFN through decreased seed dormancy and late maturity amylase production (Auld & Paulsen, 2003; Lunn et al., 2002; Mares & Mrva, 2014). Our results suggest that regions of the UK that are currently high risk for HFN due to pre‐harvest sprouting will benefit from projected warmer, drier summers, on average. However, the East of England, which currently experiences relatively high temperatures and low rainfall, and lower HFN risk, may be negatively impacted by further increased temperatures during grain development.

Specific weight has previously been positively associated with dry and sunny weather during grain filling and these weather trends are indirectly predictable on seasonal timescales based on the North Atlantic Oscillation (NAO) over winter months of the same year (Atkinson et al., 2005). This is contrary to the negative effects found here for specific weight for climate change impacts of hotter and dryer weather during grain filling in June and July, which is also consistent with negative effects found for heat and drought in hotter climates than the UK (Hernández‐Espinosa et al., 2018). However, climate change effects also include increased rainfall on average over winter and up until May when flowering occurs which we suggest also negatively impacts specific weight. Associations between predicted specific weight and weather variables support these trends and negative effects of projected climate change on specific weight in the majority of the UK wheat growing areas suggest that increased risk of wetter and more variable weather at flowering outweighs benefits of increased temperatures after flowering which may become negative as summer heat and drought pressures increase.

Taking a similar approach to de los Campos et al. (2020), who made predictions of a limited set of observed varieties' yield in historical year environments and locations, we were able to make predictions of a larger set of genotypes into all locations across the UK and a much larger number of future year environments based on climate model projections. Our results suggest that there is potential to use G × E genomic prediction models outlined here to select for increased climate resilience for HFN that would mitigate a significant proportion of year‐to‐year environmental weather variability, but much less so for the other quality traits. This could be explained in a number of ways. Firstly, genetic diversity among the highly selected elite wheats may be too narrow to enable large enough G × E for climate adaptation. However, suggestions that modern wheat cultivars lack environmental response diversity and climate resilience for yield (Kahiluoto et al., 2019) have been widely refuted (Piepho, 2019; Snowdon et al., 2019), and UK wheat genetic diversity has not decreased over the last century (Donini et al., 2000; Huang et al., 2007; White et al., 2008). The varieties included in this study were all UK registered varieties or advanced breeding lines, so are varieties that have already been tested and potentially selected for wide adaptability and high performance for quality traits across multiple years and regions in the UK. Integration of material from growing regions, such as southern Europe or Canada, that currently experience more severe heat and water deficit in the wheat growing season than for the projected UK climate may reveal more variation in G × E potential and usefully adapted material that was not considered in this study. Alternatively, the environmental variation among the years used in the observed trials dataset may be too narrow to generate sufficiently large G × E effects. Although the frequency of extreme weather events is expected to increase due to climate change, the projected change in the projected climatological average of weather covariates rarely exceed the range of environments in the observed trials dataset. Coordinated testing of a broader diversity of genotypes in a broader diversity of growing environments, such as exchange of breeding materials between breeding programmes across continents or integrating non‐elite genetic resources, may uncover potentially useful G × E adaptations using the models and approaches validated here.

We emphasise that more substantial and immediate climate change impacts will occur in more marginal wheat growing regions at lower latitudes than in the UK (Harkness et al., 2020; Zhao et al., 2017). In many ways, the UK wheat situation is in a least bad case scenario. There is therefore an urgency to apply approaches such as developed here to other crops and regions of the world as a priority, given the speed of climate change and the timescales required for variety selection and development in plant breeding programmes. The integration of enviromic characterisation of long‐term field trials and climate projection datasets with quantitative genetics models to predict future G × E will be highly relevant to breeding climate resilient crop varieties globally, enabling crop breeders to pre‐emptively select climate resilient varieties for changing environments.

## Supporting information


Supplementary figures

Table S1

## Data Availability

The trial data that support the findings of this study are available with permission from the Agriculture and Horticulture Development Board (AHDB) and the British Society of Plant Breeders Limited (BSPB) at https://ahdb.org.uk/knowledge‐library/recommended‐lists‐for‐cereals‐and‐oilseeds‐rl‐harvest‐results‐archive. Environmental weather data are freely available for observed interpolated monthly variables at https://catalogue.ceda.ac.uk/uuid/652cea3b8b4446f7bff73be0ce99ba0f and for UKCP18 climate change projections at https://ukclimateprojections‐ui.metoffice.gov.uk/products. Soil covariable properties are freely available from https://soilgrids.org/ but were extracted for specific Latitude and Longitude coordinates. R scripts for extraction of soil variables as well as for fitting and cross validation of G × E prediction models are available at https://doi.org/10.5281/zenodo.7407097.
