# Supplementary material for: Prediction of near‐term climate change impacts on UK wheat quality and the potential for adaptation through plant breeding
Source: Glob Chang Biol. 2022 Dec 23;29(5):1296–313. doi: 10.1111/gcb.16552 (PMC10108302; doi:10.1111/gcb.16552)
Supplement: Supplementary file 1 — Supplementary figures [file GCB-29-1296-s001.pdf]

SUPPLEMENTARY FIGURES

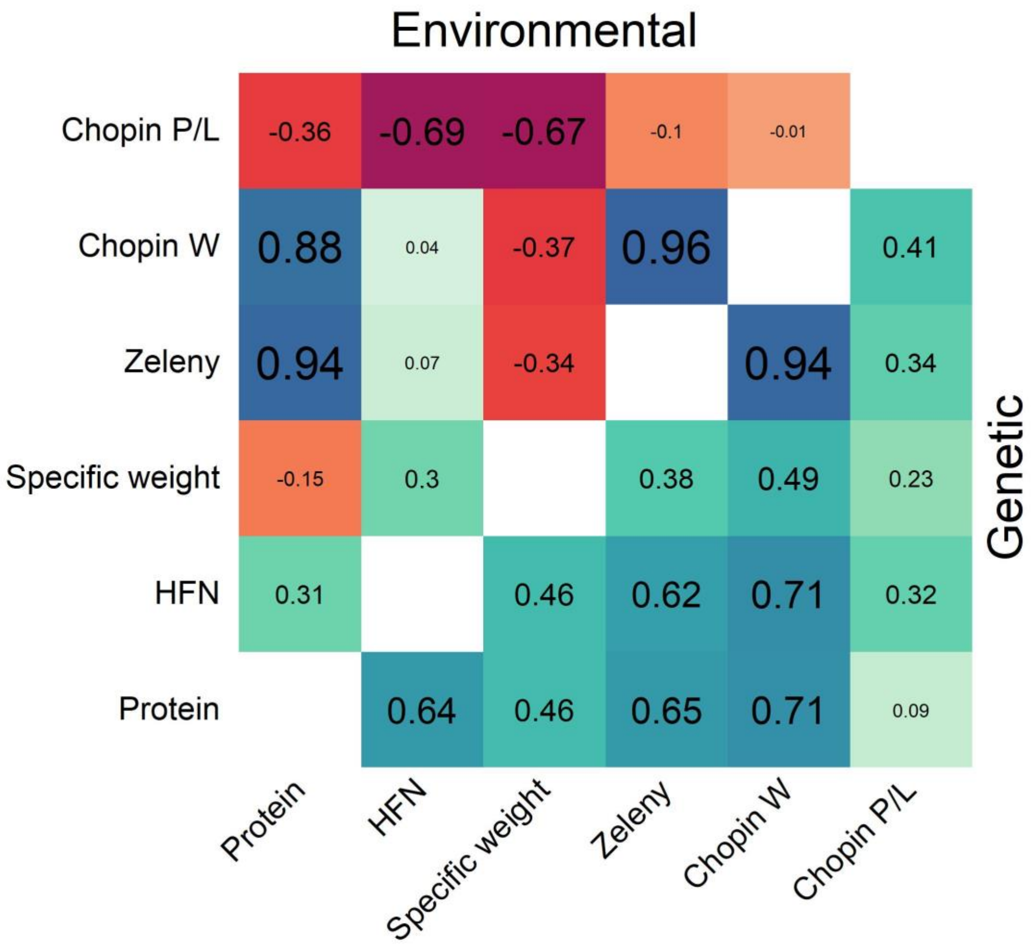

Supplementary Figure S1. Estimates of genetic and environmental correlations among quality traits in multi environment trials. Environmental and genetic correlations are shown above and below the diagonal respectively and darker red and blue colours indicate stronger negative and positive correlations respectively.

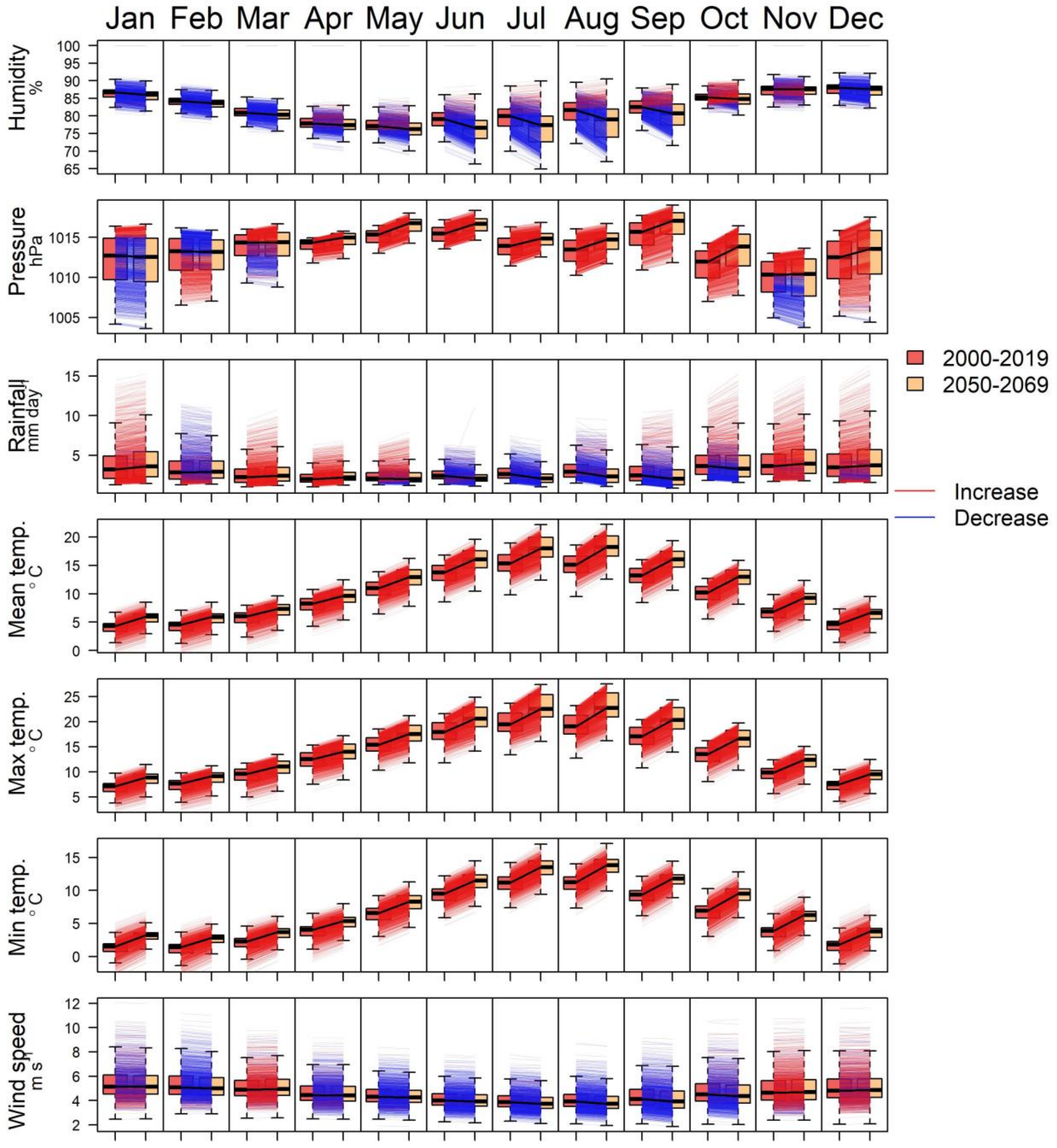

Supplementary Figure S2. Boxplots indicating the differences in each monthly weather variable between the mean observed values between 2000 and 2019 compared to the projected mean climate between 2050 to 2069 under an RCP8.5 high emissions scenario. Lines between boxplots connect values for each 12 km grid box representative of the UK land area between the observed average climate and the projected future climate.

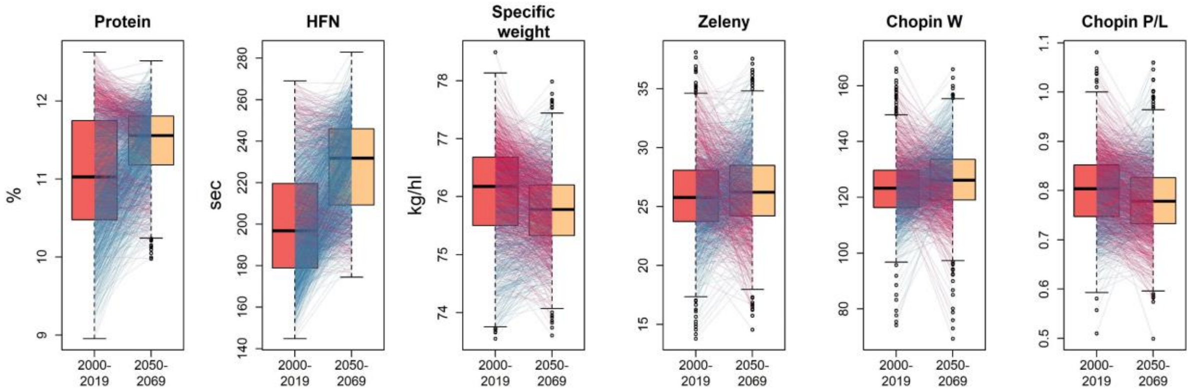

Supplementary Figure S3. Boxplots show the distributions of trait variation in the observed average (2000 - 2019) and RCP8.5 future climate conditions of (2050 – 2069). Connecting lines link each UK grid square location. Red lines indicate a decreasing trend while blue lines indicate an increase.

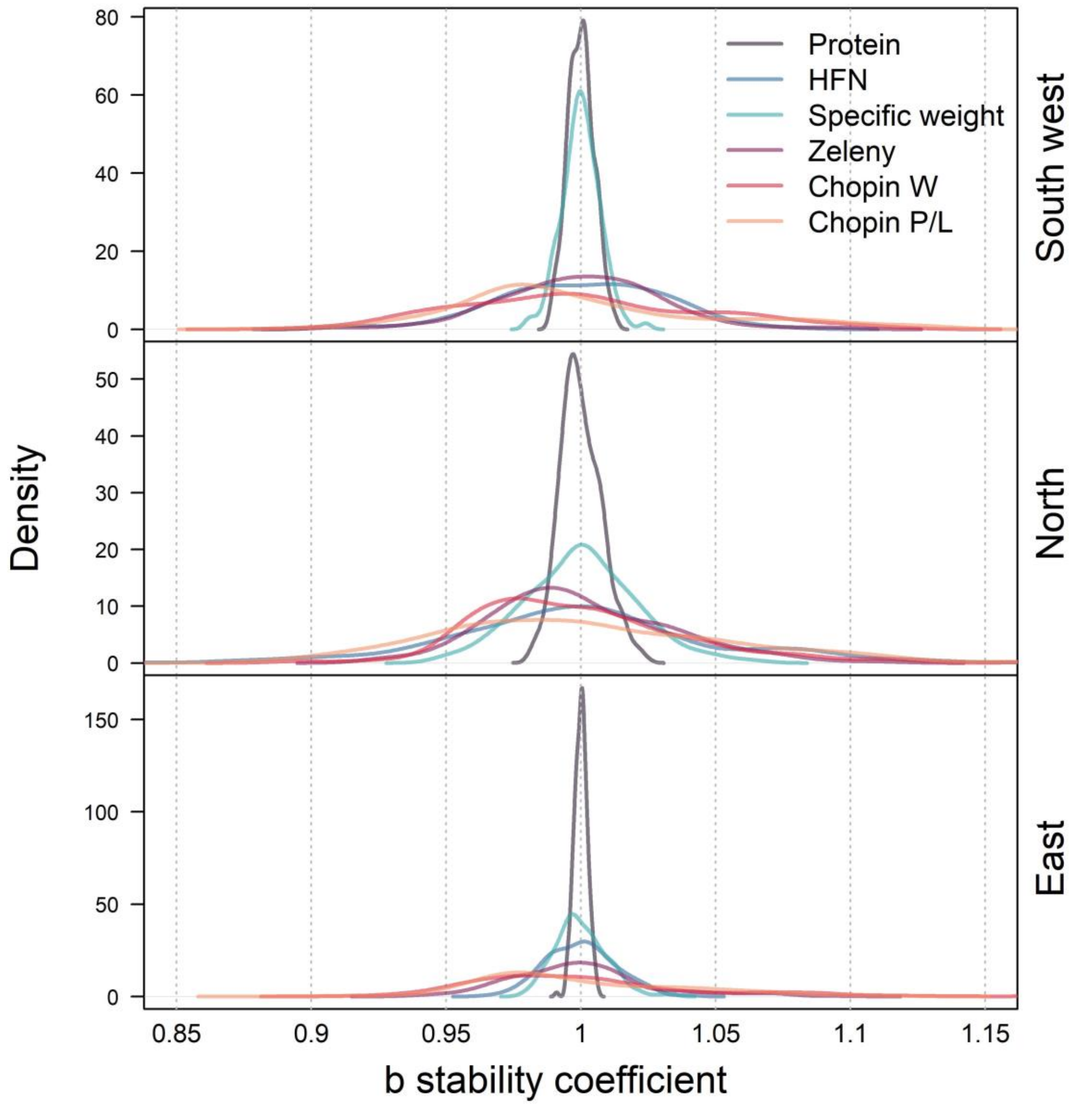

Supplementary Figure S4. Variation in b stability coefficients from Finlay Wilkinson stability analysis across all traits at three locations that were predicted to have contrasting climate change effects.

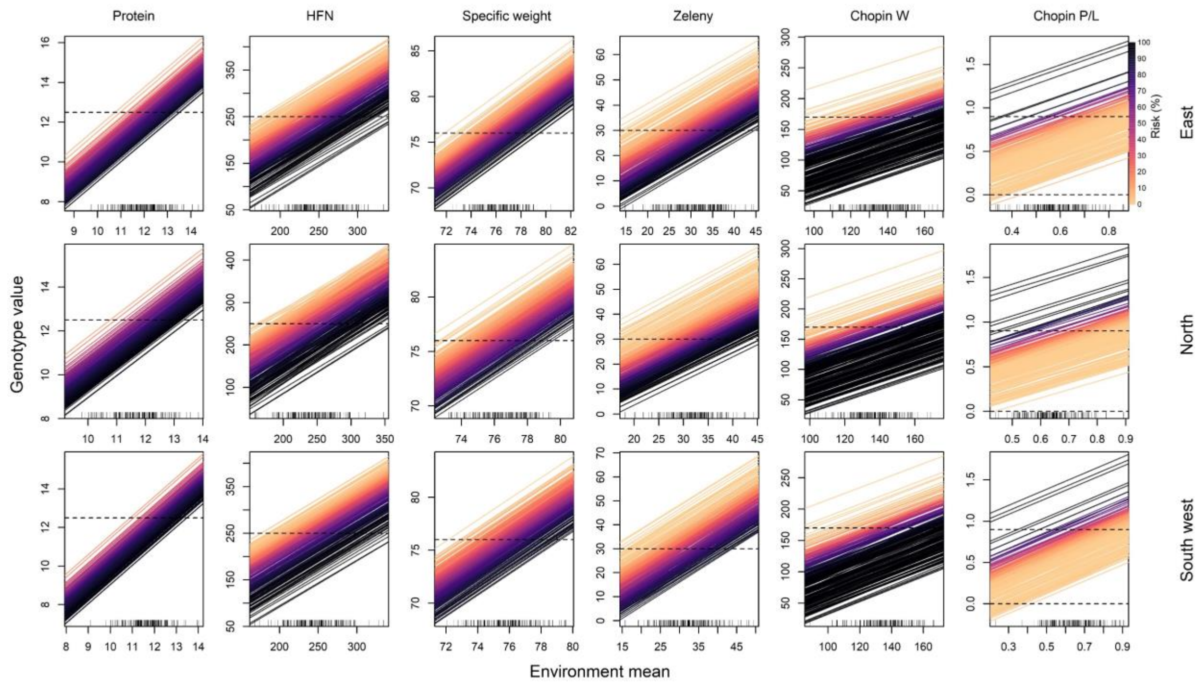

Supplementary Figure S5. Joint regression (Finlay Wilkinson) stability analysis of all 240 Genotypes over 240 future year environments under projected RCP8.5 at three locations that were predicted to have contrasting climate change effects (shown in Figure 4) for all quality traits where each line represents the adaptive response of each genotype to the environmental quality gradient. Line colour indicates the risk (percentage of years) that the trait value falls outside or below the minimum defined quality criteria thresholds (horizontal dashed lines) across all simulated environments. Dashes inside the x-axes indicate the mean trait value of all genotypes in each of 240 projected year environments.
